# 6-Benz­yloxy-2-phenyl­pyridazin-3(2*H*)-one

**DOI:** 10.1107/S1600536812018776

**Published:** 2012-05-05

**Authors:** Zhi-Yu Ju, Gong-Chun Li, Chao Li, Jie Wang, Feng-Ling Yang

**Affiliations:** a College of Chemistry and Chemical Engineering, Xuchang University, Xuchang, Henan Province 461000, People’s Republic of China; b College of Chemistry and Molecular Engineering, Zhengzhou University, Zhengzhou, Henan Province 450001, People’s Republic of China

## Abstract

In the title compound, C_17_H_14_N_2_O_2_, the central pyridazine ring forms dihedral angles of 47.29 (5) and 88.54 (5)° with the benzene rings, while the dihedral angle between the benzene rings is 62.68 (6)°. In the crystal, molecules are linked by two weak C—H⋯O hydrogen bonds and three weak C—H⋯π inter­actions.

## Related literature

For applications of pyridazinone analogues as highly selective anti-HIV agents, see: Loksha *et al.* (2007 ▶), as pesticides, see: Li *et al.* (2005 ▶) and as herbicides, see: Xu *et al.* (2006 ▶). For a related structure, see: Ju *et al.* (2011 ▶).
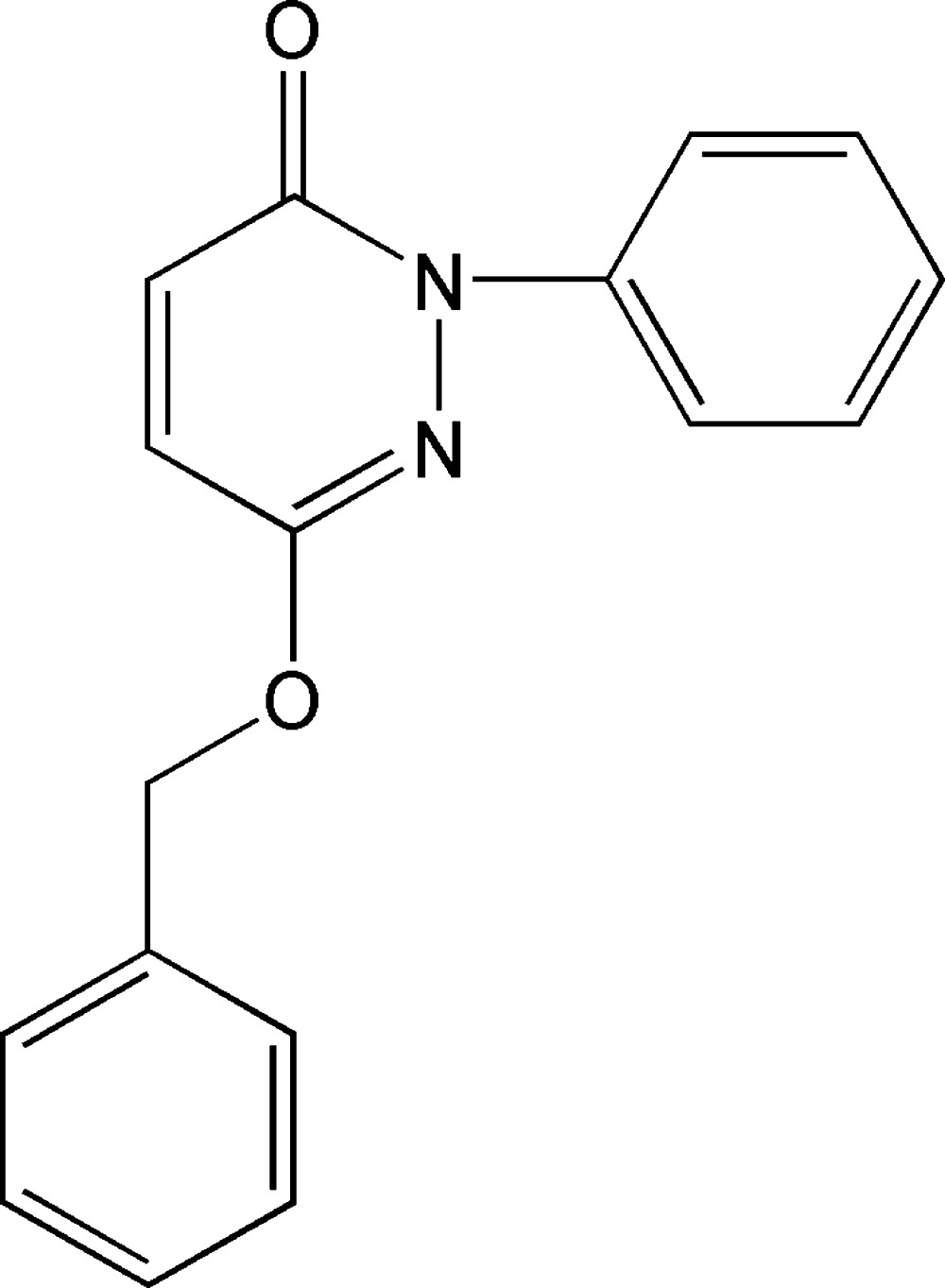


## Experimental

### 

#### Crystal data

C_17_H_14_N_2_O_2_
*M* *_r_* = 278.30
Triclinic, 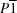

*a* = 7.390 (4) Å
*b* = 9.385 (5) Å
*c* = 10.587 (6) Å
α = 106.618 (7)°
β = 97.489 (6)°
γ = 101.098 (9)°
*V* = 676.9 (6) Å^3^
*Z* = 2
Mo *K*α radiation
μ = 0.09 mm^−1^
*T* = 113 K
0.20 × 0.18 × 0.14 mm

#### Data collection

Rigaku Saturn CCD area-detector diffractometer
Absorption correction: multi-scan (*CrystalClear*; Rigaku/MSC, 2005 ▶) *T*_min_ = 0.982, *T*_max_ = 0.987
7083 measured reflections
3167 independent reflections
2103 reflections with *I* > 2σ(*I*)
*R*_int_ = 0.037

#### Refinement

*R*[*F* ^2^ > 2σ(*F* ^2^)] = 0.036
*wR*(*F* ^2^) = 0.070
*S* = 1.02
3167 reflections
190 parameters
H-atom parameters constrained
Δρ_max_ = 0.29 e Å^−3^
Δρ_min_ = −0.21 e Å^−3^

### 

Data collection: *CrystalClear* (Rigaku/MSC, 2005 ▶); cell refinement: *CrystalClear*; data reduction: *CrystalClear*; program(s) used to solve structure: *SHELXS97* (Sheldrick, 2008 ▶); program(s) used to refine structure: *SHELXL97* (Sheldrick, 2008 ▶); molecular graphics: *SHELXTL* (Sheldrick, 2008 ▶); software used to prepare material for publication: *CrystalStructure* (Rigaku/MSC, 2005 ▶).

## Supplementary Material

Crystal structure: contains datablock(s) global, I. DOI: 10.1107/S1600536812018776/bg2457sup1.cif

Structure factors: contains datablock(s) I. DOI: 10.1107/S1600536812018776/bg2457Isup2.hkl

Supplementary material file. DOI: 10.1107/S1600536812018776/bg2457Isup3.cml

Additional supplementary materials:  crystallographic information; 3D view; checkCIF report

## Figures and Tables

**Table 1:** Hydrogen-bond geometry (Å, °) *Cg*1 and *Cg*2 are the centroids of the C1–C6 and C12–C17 rings, respectively.

*D*—H⋯*A*	*D*—H	H⋯*A*	*D*⋯*A*	*D*—H⋯*A*
C8—H8⋯O1^i^	0.95	2.54	3.389 (2)	149
C15—H15⋯O1^ii^	0.95	2.44	3.235 (2)	141
C4—H4⋯*Cg*2^iii^	0.95	2.76	3.494 (2)	135
C9—H9⋯*Cg*2^iv^	0.95	2.95	3.752 (2)	143
C13—H13⋯*Cg*1^v^	0.95	2.63	3.456 (2)	145

Symmetry codes: (i) 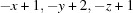; (ii) 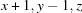; (iii) 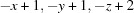; (iv) 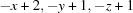; (v) 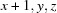.

**Fig. 1.**
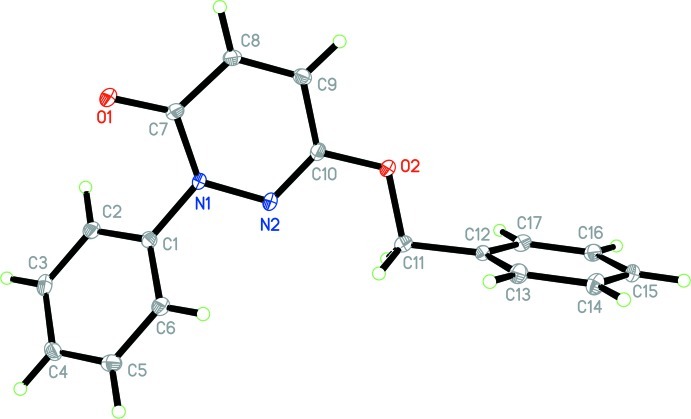
The asymmetric unit of the title compound, (I), with displacement ellipsoids drawn at the 30% probability level.

## supplementary crystallographic information

### Comment

Pyridazinones represent an important class of biologically active compounds. Recently, a substantial number of pyridazinones have been reported to possess highly-selective anti-HIV agents (Loksha *et al.*, 2007), pesticide(Li *et al.*, 2005), highly herbicidal activity (Xu *et al.*, 2006). In order to discover further biologically active Pyridazinone analogues, the title compound, (I), was synthesized, and its crystal structure determined (Fig. 1).

As a continuation of our studies on the crystal structures of Pyridazinone analogues (Ju *et al.*, 2011), we report here the synthesis and crystal structure, an ellipsoid plot of which is shown in Fig. 1. The central pyridazine ring forms dihedral angles of 47.29 (5)° and 88.54 (5)° with the two benzene rings, while the dihedral angle between the two benzene rings is 62.68 (6)°. The structure is stabilized by two weak C—H···O and three C—H···*Cg* intermolecular hydrogen bonds (Cg's: centroids of the benzene rings) (Table 1).

### Experimental

3-hydroxyl-1phenyl-6-pyridazone(0.94 g, 5 mmol), benzyl chloride(0.63 g, 5 mmol) and K_2_CO_3_ (0.69 g, 5 mmol) were added to absolute ethanol(30 ml). The mixture was stirred in the room temperature for 10 h. The suspension was filtered and the residue was washed with absolute ethanol. The title compound was recrystallized from the mother solution and single crystals of (I) were obtained by slow evaporation.

### Refinement

All H atoms were placed in calculated positions, with C—H = 0.95 Å and C—H = 0.99 Å, and included in the final cycles of refinement using a riding model, with *U*_iso_(H) = 1.2Ueq(C).

### Crystal data

**Table:**

C_17_H_14_N_2_O_2_	*Z* = 2
*M**_r_* = 278.30	*F*(000) = 292
Triclinic, *P*1	*D*_x_ = 1.365 Mg m^−^^3^
Hall symbol: -P 1	Mo *K*α radiation, λ = 0.71073 Å
*a* = 7.390 (4) Å	Cell parameters from 2420 reflections
*b* = 9.385 (5) Å	θ = 2.0–27.9°
*c* = 10.587 (6) Å	µ = 0.09 mm^−^^1^
α = 106.618 (7)°	*T* = 113 K
β = 97.489 (6)°	Prism, colorless
γ = 101.098 (9)°	0.20 × 0.18 × 0.14 mm
*V* = 676.9 (6) Å^3^	

### Data collection

**Table:**

Rigaku Saturn CCD area-detector diffractometer	3167 independent reflections
Radiation source: rotating anode	2103 reflections with *I* > 2σ(*I*)
Multilayer monochromator	*R*_int_ = 0.037
Detector resolution: 14.63 pixels mm^-1^	θ_max_ = 27.9°, θ_min_ = 2.1°
ω and φ scans	*h* = −9→9
Absorption correction: multi-scan (*CrystalClear*; Rigaku/MSC, 2005)	*k* = −12→12
*T*_min_ = 0.982, *T*_max_ = 0.987	*l* = −13→13
7083 measured reflections	

### Refinement

**Table:**

Refinement on *F*^2^	Primary atom site location: structure-invariant direct methods
Least-squares matrix: full	Secondary atom site location: difference Fourier map
*R*[*F*^2^ > 2σ(*F*^2^)] = 0.036	Hydrogen site location: inferred from neighbouring sites
*wR*(*F*^2^) = 0.070	H-atom parameters constrained
*S* = 1.02	*w* = 1/[σ^2^(*F*_o_^2^) + (0.014*P*)^2^] where *P* = (*F*_o_^2^ + 2*F*_c_^2^)/3
3167 reflections	(Δ/σ)_max_ < 0.001
190 parameters	Δρ_max_ = 0.29 e Å^−^^3^
0 restraints	Δρ_min_ = −0.21 e Å^−^^3^

### Special details

**Table:**

Geometry. All e.s.d.'s (except the e.s.d. in the dihedral angle between two l.s. planes) are estimated using the full covariance matrix. The cell e.s.d.'s are taken into account individually in the estimation of e.s.d.'s in distances, angles and torsion angles; correlations between e.s.d.'s in cell parameters are only used when they are defined by crystal symmetry. An approximate (isotropic) treatment of cell e.s.d.'s is used for estimating e.s.d.'s involving l.s. planes.
Refinement. Refinement of *F*^2^ against ALL reflections. The weighted *R*-factor *wR* and goodness of fit *S* are based on *F*^2^, conventional *R*-factors *R* are based on *F*, with *F* set to zero for negative *F*^2^. The threshold expression of *F*^2^ > σ(*F*^2^) is used only for calculating *R*-factors(gt) *etc*. and is not relevant to the choice of reflections for refinement. *R*-factors based on *F*^2^ are statistically about twice as large as those based on *F*, and *R*- factors based on ALL data will be even larger.

### Fractional atomic coordinates and isotropic or equivalent isotropic displacement parameters (Å^2^)

**Table:**

	*x*	*y*	*z*	*U*_iso_*/*U*_eq_	
O1	0.42844 (11)	0.94745 (8)	0.63170 (7)	0.0233 (2)	
O2	0.77932 (11)	0.49972 (8)	0.63235 (8)	0.0216 (2)	
N1	0.48299 (13)	0.75278 (10)	0.71052 (9)	0.0164 (2)	
N2	0.57426 (13)	0.63946 (10)	0.71641 (9)	0.0175 (2)	
C1	0.36869 (16)	0.78488 (12)	0.81087 (11)	0.0167 (3)	
C2	0.18835 (17)	0.79994 (12)	0.77449 (12)	0.0201 (3)	
H2	0.1387	0.7879	0.6836	0.024*	
C3	0.08053 (17)	0.83289 (12)	0.87255 (12)	0.0220 (3)	
H3	−0.0429	0.8447	0.8488	0.026*	
C4	0.15216 (17)	0.84853 (12)	1.00468 (12)	0.0226 (3)	
H4	0.0780	0.8708	1.0713	0.027*	
C5	0.33193 (17)	0.83158 (12)	1.03932 (12)	0.0216 (3)	
H5	0.3805	0.8414	1.1298	0.026*	
C6	0.44193 (17)	0.80031 (12)	0.94267 (11)	0.0194 (3)	
H6	0.5658	0.7896	0.9667	0.023*	
C7	0.50525 (16)	0.84005 (12)	0.62487 (11)	0.0175 (3)	
C8	0.62325 (15)	0.79457 (12)	0.52976 (11)	0.0187 (3)	
H8	0.6404	0.8461	0.4653	0.022*	
C9	0.70914 (16)	0.68125 (12)	0.53036 (11)	0.0190 (3)	
H9	0.7855	0.6509	0.4669	0.023*	
C10	0.68183 (16)	0.60737 (12)	0.63011 (11)	0.0172 (3)	
C11	0.76814 (17)	0.43746 (13)	0.74356 (11)	0.0219 (3)	
H11A	0.6432	0.3673	0.7289	0.026*	
H11B	0.7866	0.5214	0.8293	0.026*	
C12	0.91981 (16)	0.35233 (12)	0.74890 (11)	0.0178 (3)	
C13	1.09241 (17)	0.42585 (13)	0.83407 (11)	0.0218 (3)	
H13	1.1142	0.5302	0.8874	0.026*	
C14	1.23333 (17)	0.34891 (13)	0.84226 (11)	0.0230 (3)	
H14	1.3512	0.4006	0.9008	0.028*	
C15	1.20271 (17)	0.19639 (13)	0.76516 (11)	0.0217 (3)	
H15	1.2986	0.1428	0.7712	0.026*	
C16	1.03045 (17)	0.12308 (13)	0.67916 (11)	0.0217 (3)	
H16	1.0090	0.0189	0.6255	0.026*	
C17	0.89004 (17)	0.19985 (12)	0.67083 (11)	0.0206 (3)	
H17	0.7727	0.1484	0.6116	0.025*	

### Atomic displacement parameters (Å^2^)

**Table:**

	*U*^11^	*U*^22^	*U*^33^	*U*^12^	*U*^13^	*U*^23^
O1	0.0327 (5)	0.0207 (5)	0.0235 (5)	0.0139 (4)	0.0090 (4)	0.0113 (4)
O2	0.0276 (5)	0.0237 (5)	0.0227 (5)	0.0151 (4)	0.0099 (4)	0.0134 (4)
N1	0.0187 (6)	0.0172 (5)	0.0177 (5)	0.0093 (4)	0.0052 (5)	0.0083 (4)
N2	0.0189 (6)	0.0168 (5)	0.0198 (5)	0.0085 (4)	0.0041 (5)	0.0074 (4)
C1	0.0200 (7)	0.0130 (6)	0.0188 (7)	0.0044 (5)	0.0066 (5)	0.0062 (5)
C2	0.0241 (7)	0.0176 (6)	0.0199 (7)	0.0065 (5)	0.0040 (6)	0.0075 (5)
C3	0.0196 (7)	0.0181 (6)	0.0314 (8)	0.0078 (5)	0.0071 (6)	0.0096 (5)
C4	0.0290 (8)	0.0176 (7)	0.0243 (7)	0.0080 (6)	0.0129 (6)	0.0067 (5)
C5	0.0271 (8)	0.0199 (7)	0.0169 (7)	0.0034 (6)	0.0043 (6)	0.0061 (5)
C6	0.0202 (7)	0.0180 (6)	0.0209 (7)	0.0050 (5)	0.0038 (6)	0.0077 (5)
C7	0.0201 (7)	0.0170 (6)	0.0158 (6)	0.0044 (5)	0.0016 (5)	0.0067 (5)
C8	0.0224 (7)	0.0195 (6)	0.0165 (6)	0.0056 (5)	0.0052 (6)	0.0081 (5)
C9	0.0201 (7)	0.0215 (7)	0.0163 (7)	0.0053 (5)	0.0056 (5)	0.0062 (5)
C10	0.0185 (7)	0.0164 (6)	0.0170 (6)	0.0067 (5)	0.0012 (5)	0.0051 (5)
C11	0.0255 (8)	0.0243 (7)	0.0223 (7)	0.0104 (6)	0.0078 (6)	0.0132 (5)
C12	0.0210 (7)	0.0199 (6)	0.0185 (7)	0.0098 (5)	0.0081 (6)	0.0102 (5)
C13	0.0270 (8)	0.0166 (6)	0.0238 (7)	0.0074 (6)	0.0066 (6)	0.0074 (5)
C14	0.0194 (7)	0.0240 (7)	0.0257 (7)	0.0058 (6)	0.0014 (6)	0.0090 (6)
C15	0.0249 (7)	0.0259 (7)	0.0229 (7)	0.0151 (6)	0.0095 (6)	0.0128 (6)
C16	0.0322 (8)	0.0167 (6)	0.0186 (7)	0.0101 (6)	0.0071 (6)	0.0056 (5)
C17	0.0219 (7)	0.0216 (7)	0.0187 (7)	0.0052 (5)	0.0020 (5)	0.0080 (5)

### Geometric parameters (Å, º)

**Table:**

O1—C7	1.2379 (14)	C8—C9	1.3405 (15)
O2—C10	1.3526 (14)	C8—H8	0.9500
O2—C11	1.4611 (14)	C9—C10	1.4343 (16)
N1—N2	1.3758 (13)	C9—H9	0.9500
N1—C7	1.3899 (14)	C11—C12	1.5006 (16)
N1—C1	1.4429 (14)	C11—H11A	0.9900
N2—C10	1.2967 (14)	C11—H11B	0.9900
C1—C2	1.3836 (17)	C12—C13	1.3862 (16)
C1—C6	1.3850 (17)	C12—C17	1.3907 (16)
C2—C3	1.3911 (16)	C13—C14	1.3839 (16)
C2—H2	0.9500	C13—H13	0.9500
C3—C4	1.3851 (17)	C14—C15	1.3875 (17)
C3—H3	0.9500	C14—H14	0.9500
C4—C5	1.3829 (17)	C15—C16	1.3875 (16)
C4—H4	0.9500	C15—H15	0.9500
C5—C6	1.3896 (16)	C16—C17	1.3799 (16)
C5—H5	0.9500	C16—H16	0.9500
C6—H6	0.9500	C17—H17	0.9500
C7—C8	1.4432 (16)		
			
C10—O2—C11	115.71 (9)	C8—C9—H9	121.1
N2—N1—C7	125.17 (10)	C10—C9—H9	121.1
N2—N1—C1	113.91 (9)	N2—C10—O2	119.85 (10)
C7—N1—C1	120.75 (10)	N2—C10—C9	124.22 (12)
C10—N2—N1	116.73 (10)	O2—C10—C9	115.93 (11)
C2—C1—C6	120.99 (11)	O2—C11—C12	107.55 (9)
C2—C1—N1	119.79 (11)	O2—C11—H11A	110.2
C6—C1—N1	119.22 (11)	C12—C11—H11A	110.2
C1—C2—C3	119.21 (11)	O2—C11—H11B	110.2
C1—C2—H2	120.4	C12—C11—H11B	110.2
C3—C2—H2	120.4	H11A—C11—H11B	108.5
C4—C3—C2	120.34 (12)	C13—C12—C17	119.09 (12)
C4—C3—H3	119.8	C13—C12—C11	119.69 (11)
C2—C3—H3	119.8	C17—C12—C11	121.21 (11)
C5—C4—C3	119.82 (11)	C14—C13—C12	120.71 (11)
C5—C4—H4	120.1	C14—C13—H13	119.6
C3—C4—H4	120.1	C12—C13—H13	119.6
C4—C5—C6	120.45 (12)	C13—C14—C15	120.09 (12)
C4—C5—H5	119.8	C13—C14—H14	120.0
C6—C5—H5	119.8	C15—C14—H14	120.0
C1—C6—C5	119.19 (12)	C14—C15—C16	119.22 (12)
C1—C6—H6	120.4	C14—C15—H15	120.4
C5—C6—H6	120.4	C16—C15—H15	120.4
O1—C7—N1	121.54 (11)	C17—C16—C15	120.69 (12)
O1—C7—C8	123.94 (10)	C17—C16—H16	119.7
N1—C7—C8	114.53 (11)	C15—C16—H16	119.7
C9—C8—C7	121.43 (11)	C16—C17—C12	120.20 (12)
C9—C8—H8	119.3	C16—C17—H17	119.9
C7—C8—H8	119.3	C12—C17—H17	119.9
C8—C9—C10	117.73 (11)		
			
C7—N1—N2—C10	−3.67 (15)	N1—C7—C8—C9	−2.77 (16)
C1—N1—N2—C10	−178.83 (9)	C7—C8—C9—C10	−0.74 (16)
N2—N1—C1—C2	−134.36 (10)	N1—N2—C10—O2	178.41 (9)
C7—N1—C1—C2	50.24 (14)	N1—N2—C10—C9	−0.47 (16)
N2—N1—C1—C6	45.70 (13)	C11—O2—C10—N2	−5.75 (15)
C7—N1—C1—C6	−129.70 (12)	C11—O2—C10—C9	173.22 (9)
C6—C1—C2—C3	0.78 (16)	C8—C9—C10—N2	2.57 (17)
N1—C1—C2—C3	−179.16 (9)	C8—C9—C10—O2	−176.35 (9)
C1—C2—C3—C4	−0.80 (16)	C10—O2—C11—C12	−166.04 (9)
C2—C3—C4—C5	0.13 (16)	O2—C11—C12—C13	94.70 (12)
C3—C4—C5—C6	0.58 (16)	O2—C11—C12—C17	−86.01 (13)
C2—C1—C6—C5	−0.09 (16)	C17—C12—C13—C14	−0.35 (17)
N1—C1—C6—C5	179.85 (9)	C11—C12—C13—C14	178.96 (10)
C4—C5—C6—C1	−0.60 (16)	C12—C13—C14—C15	−0.22 (18)
N2—N1—C7—O1	−174.84 (10)	C13—C14—C15—C16	0.68 (17)
C1—N1—C7—O1	0.01 (17)	C14—C15—C16—C17	−0.59 (17)
N2—N1—C7—C8	5.19 (16)	C15—C16—C17—C12	0.02 (17)
C1—N1—C7—C8	−179.96 (9)	C13—C12—C17—C16	0.44 (17)
O1—C7—C8—C9	177.26 (11)	C11—C12—C17—C16	−178.85 (10)

### Hydrogen-bond geometry (Å, º)

Cg1 and Cg2 are the centroids of the C1–C6 and C12–C17 rings, respectively.

**Table:**

*D*—H···*A*	*D*—H	H···*A*	*D*···*A*	*D*—H···*A*
C8—H8···O1^i^	0.95	2.54	3.389 (2)	149
C15—H15···O1^ii^	0.95	2.44	3.235 (2)	141
C4—H4···*Cg*2^iii^	0.95	2.76	3.494 (2)	135
C9—H9···*Cg*2^iv^	0.95	2.95	3.752 (2)	143
C13—H13···*Cg*1^v^	0.95	2.63	3.456 (2)	145

Symmetry codes: (i) −*x*+1, −*y*+2, −*z*+1; (ii) *x*+1, *y*−1, *z*; (iii) −*x*+1, −*y*+1, −*z*+2; (iv) −*x*+2, −*y*+1, −*z*+1; (v) *x*+1, *y*, *z*.
